# Supplementary material for: Complexome profiling of the Chlamydomonas psb28 mutant reveals TEF5 as an early PSII assembly factor
Source: Plant Cell. 2025 Mar 17;37(6):koaf055. doi: 10.1093/plcell/koaf055 (PMC12164590; doi:10.1093/plcell/koaf055)
Supplement: koaf055_Supplementary_Data [file koaf055_supplementary_data.zip › psb28_interactive.zip!/plots/intensity/qualityPlots/peptides/_Cre03.g167400.t1.2.html]

### Cre03.g167400.t1.2

Trivial name:   
SeqCoverage [median]: 5.40  
Mass: 33.54 kDa  
  
 

| Sequence | MissedCleavage | Mass [Da] | wtIntensity | muIntensity | Score |
| --- | --- | --- | --- | --- | --- |
| ITSRPDELR | 1 | 1085.58 | 412.63 | 375.35 | 45.16 |
| LGQQEGAK | 0 | 829.43 | 212.63 | 0.00 | 107.90 |
|  |  |  | 625.3 | 375.4 |  |
